# Direct-write, highly aligned chitosan-poly(ethylene oxide) nanofiber patterns for cell morphology and spreading control

**DOI:** 10.1186/1556-276X-8-97

**Published:** 2013-02-22

**Authors:** Yiin Kuen Fuh, Sheng Zhan Chen, Zhe Yu He

**Affiliations:** 1 Department of Mechanical Engineering, National Central University, Taoyuan County, 32001, Taiwan; 2 Institute of Energy Engineering, National Central University, Taoyuan County, 32001, Taiwan

**Keywords:** Chitosan nanofibers, Near-field electrospinning, Direct-write patterns

## Abstract

Near-field electrospinning has been demonstrated to be able to achieve direct-write and highly aligned chitosan nanofibers (CNF) with prescribed positioning density. Cell spreading in preferential direction could be observed on parallel-aligned nanofibers, and the CNF patterns were capable of guiding cell extension when the distances between them are 20 and 100 μm, respectively. Alignment of the cells was characterized according to their elongation and orientation using the fast Fourier transform data and binary image analysis. Parallel CNF indicates that the alignment values sequentially increased as a function of positioning density such that incrementally more aligned cells were closely related to the increasing CNF positioning density. These maskless, low-cost, and direct-write patterns can be facily fabricated and will be a promising tool to study cell-based research such as cell adhesion, spreading, and tissue architecture.

## Background

Physicochemical properties of scaffold materials are found to be critical in regulating cell behaviors and cell-material interaction in tissue engineering. For example, altering the various substances of different chemical compositions, wettability, and topography is the most common practice to control cell responses in the past decades [1,2]. Extracellular matrix consist of nanoscaled fibrous morphology has been considered beneficial in tissue regeneration due to its bio-mimicking characteristics [3]. On the other hand, behaviors of cells on conducting polymers such as polypyrrole (PPy) and polyaniline have demonstrated enhanced growth and differentiation of cardiac myoblasts [4,5], neurons [6,7], and skeletal muscle cells [8,9] because of direct electrical stimulation or electroactivity effect. In terms of biocompatible materials, chitosan is widely adopted due to its unique properties such as being naturally nontoxic, biodegradable, and antimicrobial [10]. It has been demonstrated as a promising scaffolding material in tissue engineering [11].

Electrospinning is a simple yet versatile technique for producing nanofibers. An electrically driven jet initiating from a polymeric solution through so-called Taylor cones can deposit a rich variety of polymers, composites, and ceramics with diameter ranging from tens of nanometers to few microns [12]. Previously, chitosan solutions blended with poly(ethylene oxide) (PEO) and poly(vinyl alcohol) (PVA) have been successfully electrospun [13] via a conventional electrospinning process. However, the chaotic nature of conventional electrospinning process will result in instability of the polymer jet and deposit nanofibers in a disordered and random fashion [14]. Continuous near-field electrospinning (NFES) was recently developed as a favorable technology due to its precise location control for nanofiber deposition and sophisticated patterns [15,16]. Fundamentally, when the needle-to-collector distance implemented a significant reduction from several centimeters to few millimeters, the applied bias voltage can be reduced to few hundreds of volts. A recent application of direct-write, well-aligned chitosan-poly(ethylene oxide) nanofibers deposited via near-field electrospinning was carried out to exhibit excellent deposition of aligned nanofiber patterns [17]. Electrospun nanofiber-based scaffolding systems were found to be able to achieve good cell alignment [18,19]. The cell interaction between the prescribed microscale patterns of nanofibers and macroscale specimen was experimentally observed with particular focus on cellular alignment and associated tissue architecture [20]. Furthermore, microfluidic synthesis of pure chitosan microfibers without any chemical additive for bio-artificial liver chip applications was proposed, and the chemical, mechanical, and diffusion properties of pure chitosan microfibers were analyzed [21]. Micropatterns of double-layered, multifunctional nanofiber scaffolds with dual functions of cell patterning and metabolite detection have been developed consisting of multiple layers of nanofiber scaffolds and nanofiber-incorporated poly(ethylene glycol) hydrogels [22]. Recent micro/nano technologies have opened up emerging interests to investigate relevant biological effects. For example, new nanomaterial-based assays are developed to quantitatively assess dose effect issues and related size dependence response [23]. Furthermore, under the action of rare earth oxide nanoparticle with respect to the nature of cytotoxin, cell proliferation and apoptosis are presented in [24]. In this paper, NFES was utilized to achieve direct-write and highly aligned chitosan nanofiber (CNF) with prescribed positioning density. The controlled and well-aligned CNFs are used to investigate cell spreading phenomena and related issues of cellular biocompatibility. The fundamental issues of cell spreading and extension guiding in a preferential direction are experimentally performed on parallel-aligned and grid patterns for the purpose of better realization of the ability to manipulate cellular architecture.

## Methods

### Materials

Chitosan from crab shells with 85% deacetylation (Mw = 50 to 190 kDa) was purchased from Sigma Chemical Co (St. Louis, MO, USA). PEO (Mw = 900 kDa; Triton X-100™) was provided by Acros Co. (Geel, Belgium), and dimethylsulfoxide (DMSO) was obtained from Tedia Co. (Fairfield, OH, USA). All reagents were used as received from the manufacturer without further purification.

### Preparation of stock solutions for electrospinning

Chitosan solution (5%) and 1% PEO solution were first prepared separately by dissolving chitosan in 0.5 M acetic acid, then vacuumed in an oven at 0.8 Torr to remove air bubbles [17]. Solutions containing 0.5 wt.% of Triton X-100™ and 5 to 10 wt.% of DMSO were mixed with the chitosan/PEO solutions, and the mixtures were again stirred for 16 h and vacuumed to remove air bubbles before use.

### Polypyrrole substrates

Soluble PPy was synthesized chemically using ammonium persulfate (APS) as an oxidant and a dopant. Pyrrole of 0.3 mol and 1:50 ratio of APS and pyrrole solution were mixed with 500 ml of distilled water. The solution was spin-cast on a polystyrene Petri dish to obtain a PPy film [25], and the electrical conductivity was measured to be 7.25 kΩ/square using the four-point probe method.

### NFES setup

The stock solution for electrospinning was fed into a 1-ml disposable syringe fitted with a 0.4-mm-wide needle tip, the applied electrostatic voltage was in the range of 800 to 1,000 V (AU-1592, Matsusada Precision Inc., Kusatsu, Japan), and the distance between the syringe tip and the grounded collector was 500 μm. The substrate was mounted onto a programmable XY stage (Yokogawa Inc., Tokyo, Japan), controlled by a personal computer, which allows movement of the sample during nanofiber deposition. The experiment was carried out at room temperature and atmospheric pressure.

### Cell culture, adhesion, and spreading

Human embryonic kidney cells (HEK 293T) were cultured in 25-cm^2^ flasks in Dulbecco's modified Eagle medium containing 10% fetal bovine serum. The cell suspension was added to each nanofiber pattern in a PPy-modified polystyrene Petri dish and cultured in an incubator at 37°C with 5% CO_2_.

In order to seed HEK 293T cells onto the CNF, a confluent monolayer of cells was trypsinized and centrifuged at 1,000 rpm for 4 min. After supernatant removal and re-suspension in fresh culture medium, cells were transferred to a PPy-modified polystyrene Petri dish.

### Quantification of HEK 293T alignment

Fast Fourier transform (FFT) analysis was used to characterize the alignment of HEK 293T as a function of the positioning density of CNF previously [20]. Relative alignment of CNF in electrospun scaffolds can be quantitatively evaluated via FFT analysis. FFT was conducted using ImageJ software (NIH, Maryland, USA) [26] supported by an Oval Profile plug-in. Bright-field microscopic images of cells in a grayscale 8-bit TIF format were initially cropped to 1,024 × 1,024 pixels and imported into the Oval Profile plug-in for detailed FFT analysis. Typically, the degree of alignment can be reflected by the height and overall shape of the peak. The principal angle of HEK 293T orientation can be represented by the position of the peak.

## Results and discussion

### Electrospinning

**Figure 1:**
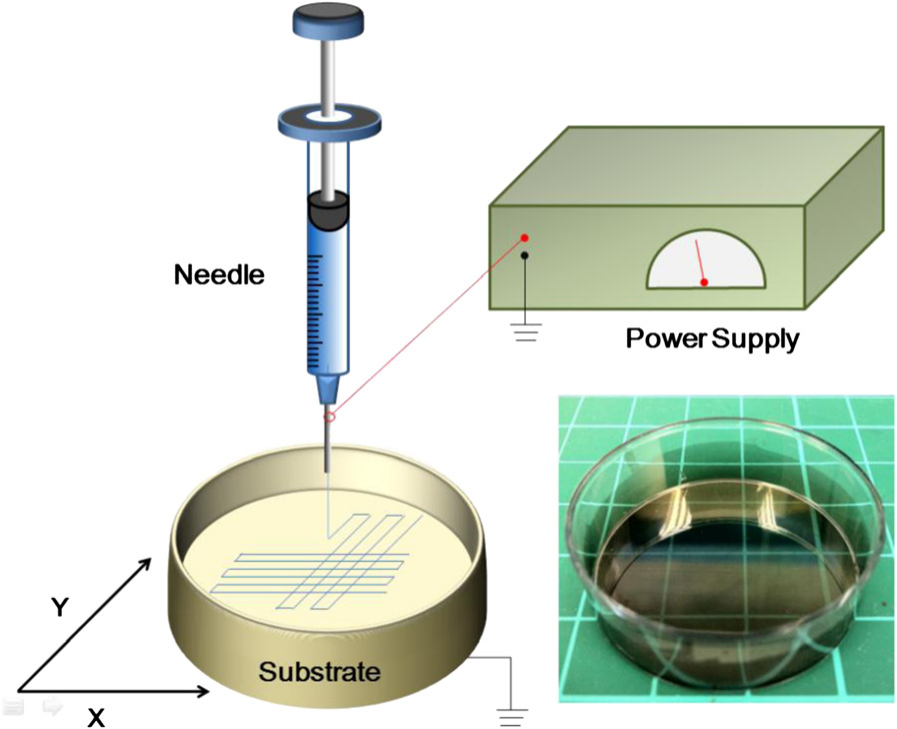
**The experimental setup.** Schematic view of the experimental setup using NFES process and direct-write patterns on PPy-modified polystyrene Petri dish via the spin-cast method exhibiting electrical conductivity of 7.25 kΩ/square. Average diameter = 431.1 nm.

**Figure 2:**
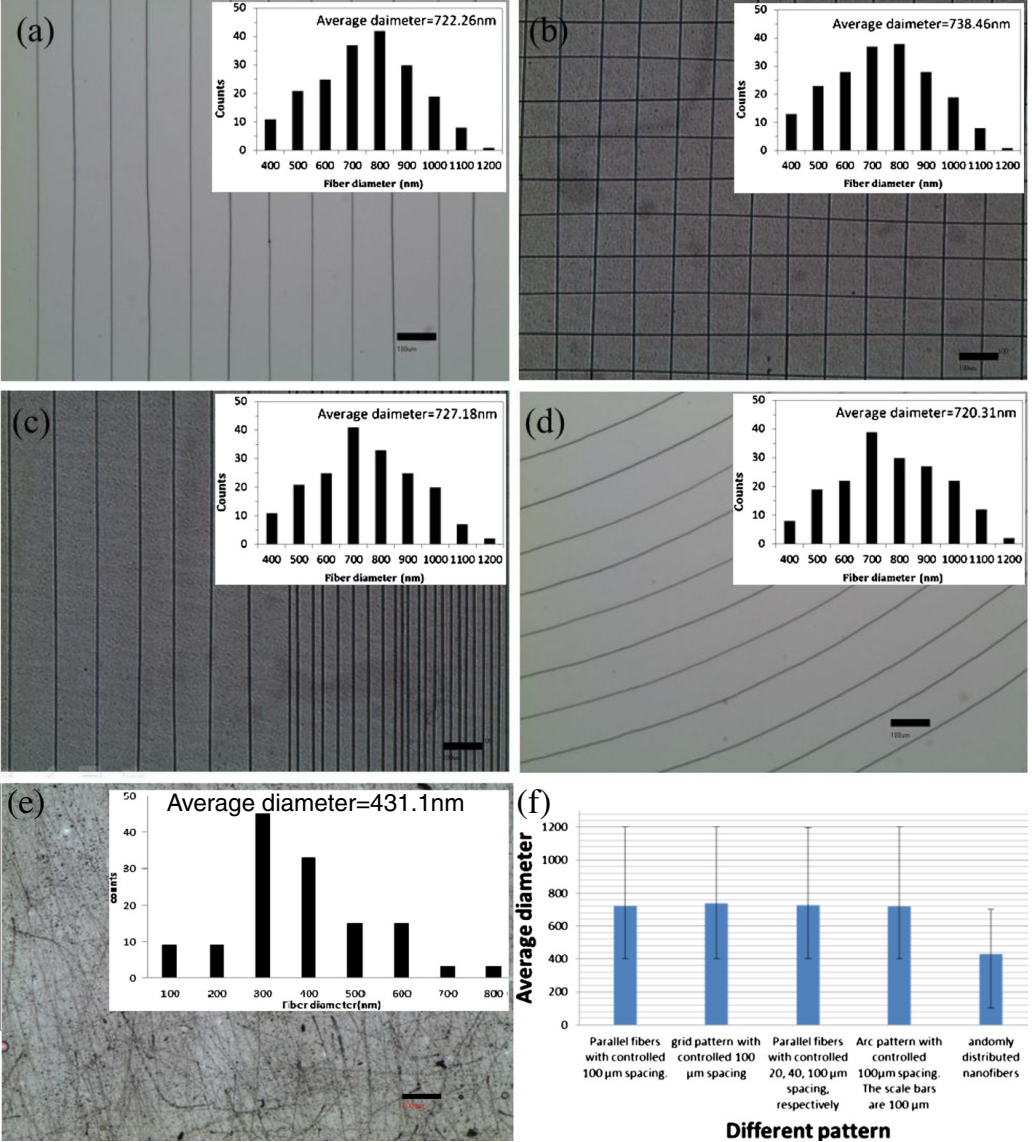
**Experiments showing controllability of NFES for chitosan/PEO fibers.** (**a**) Parallel fibers with controlled 100-μm spacing. (**b**) A grid pattern with controlled 100-μm spacing. (**c**) Parallel fibers with controlled 20-, 40-, and 100-μm spacing, respectively. (**d**) Arc pattern with controlled 100-μm spacing. The scale bars are 100 μm. (**e**) Randomly distributed nanofibers deposited via conventional electrospinning at 20 cm/s with 15 kV. (**f**) The average fiber diameter with standard deviation for the patterns of (**a**), (**b**), (**c**), (**d**), and (**e**).

The schematic of the NFES experimental setup is shown in Figure 1. Due to the near-field effect of reduced needle-to-collector distance at 500 μm, the applied voltage was 0.8 kV, which corresponds to the electric field of 1.6 × 10^6^ V/m. This was equivalent to the field strength of the reported NFES at 1.2 × 10^6^ V/m [27]. The XY stage movement speed was set at 20 cm/s. Controllability of the prescribed parallel and arc patterns of CNF is presented in Figure 2. Parallel arrays of CNF with controlled 100-μm spacing were shown in Figure 2a, and the inset shows the diameter distribution with an average value at 722.26 nm. Controlled deposition of the prescribed grid patterns at a specified distance of 100 μm was shown in Figure 2b, and the inset shows that the average diameter of the CNF was 738.46 nm. Nanofiber-induced gradient at incremental spacings of 20, 40, and 100 μm, respectively, was demonstrated in Figure 2c, and the average diameter of the CNF was 727.18 nm. These maskless, low-cost, and direct-write patterns can be easily fabricated and will be used to study cell-based research such as cell adhesion and spreading. In addition, Figure 2d demonstrates multiple arc shapes with an average diameter of 720.31 nm and separation increment of 100 μm. Above-average diameters can be well controlled in the range of 720.31 to 738.46 nm, and variation was less than 2.5%. This was a remarkable achievement even though the NFES parameters were kept the same. Moreover, scalability and preparation of well-ordered nanostructures having a length of up to several millimeters can be facily realized. Regardless of the intricacy of the pattern, the technique of balancing the speed of the XY stage and the electrospinning deposition rate was essential for continuous operation of the NFES process. Figure 2e presents the randomly distributed nanofibers deposited via conventional electrospinning, and Figure 2f shows the average fiber diameter with standard deviation for the prescribed patterns in Figure 2a,b,c,d,e. It is experimentally observed that NFES has average fiber diameters in the range of 720 to 738 nm irrespective of the prescribed patterns and spacings, while conventional electrospinning exhibits a smaller average fiber diameter of 431 nm. Fine fiber formation from conventional electrospinning is mainly achieved by stretching and acceleration of jets in high electric field [28], which the NFES counterpart aimed to reduce for better controllability of fiber deposition.

### Integrity of nanofibrous structure in water

**Figure 3:**
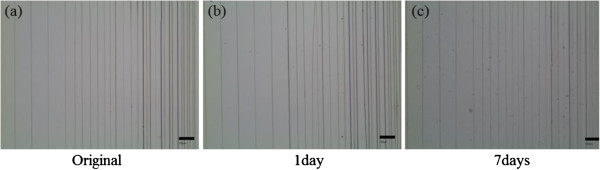
**OM images of CNF.** Morphologies of parallel CNF patterns (**a**) before and after immersion in DI water at room temperature for (**b**) 1 and (**c**) 7 days, respectively.

Since PEO is highly soluble in water [29], it is of practical interest to study the integrity of the nanofibrous structure in water. As shown in the optical images (OM) images in Figure 3, the CNF with our solution shows no significant change in the morphology of the parallel patterns after immersion in deionized (DI) water at room temperature for the periods of 1 and 7 days, respectively. It is experimentally proven that the integrity of the fibrous structure using 5% chitosan and 1% PEO can be well retained in water.

### Cell viability, adhesion, and spreading

**Figure 4:**
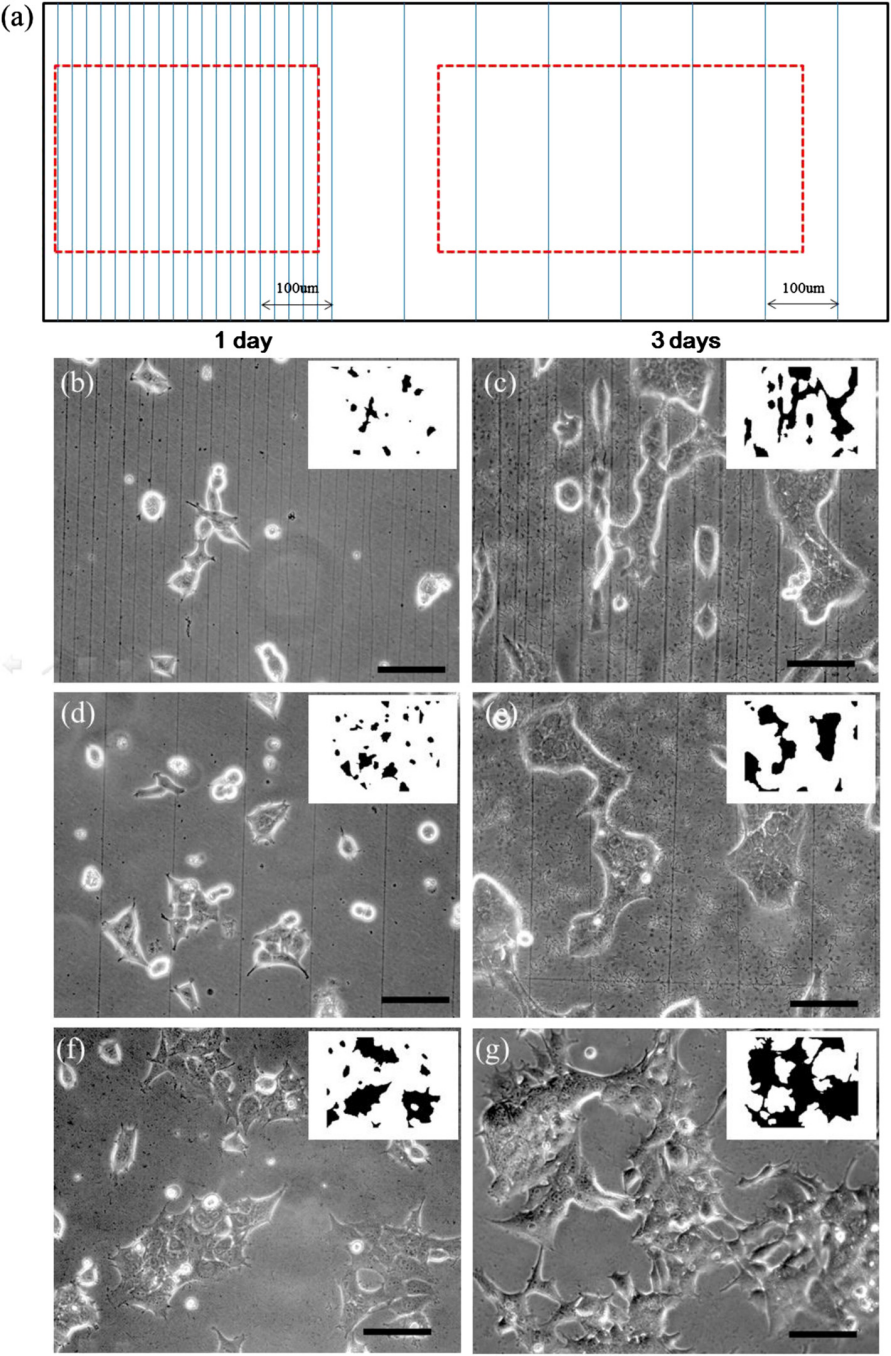
**OM images of HEK 293T cells seeded on PPy substrate covered with aligned CNF.** (**a**) Schematic illustration of the NFES-aligned CNF of different positioning densities. (**b**, **c**) Approximately 50 fibers/mm^2^ (20 μm), (**d**, **e**) approximately 10 fibers/mm^2^ (100 μm), and (**f**, **g**) cells seeded on nanofiber-free solid substrate. The smaller images at the right upper corner are shown to reveal the orientation of the cells (not on scale). The scale bars are 100 μm.

Figure 4 shows the OM images of cell viability, adhesion, and spreading on various aligned CNFs. Figure 4a is a schematic illustration of the NFES-aligned CNF deposited on the same PPy substrate with different positioning densities with a controlled 20-μm (left) and 100-μm spacing (right), respectively. The advantage of using the same cell cultivation condition on the same substrate can be applied with two different nanofiber densities. Fiber densities in Figure 4b,c are approximately 50 fibers/mm^2^ (20-μm spacing), and in Figure 4d,e, approximately 10 fibers/mm^2^ (100-μm spacing). Figure 4f,g shows cells seeded on nanofiber-free substrate for the purpose of comparison. The smaller images at the right upper corner are shown to reveal the orientation of the cells.

HEK 293T cell was selected in the present study to assess cell viability and spreading on aligned CNF. HEK 293T cells are often used as an *in vitro* model to assess cytotoxicity and has been well characterized for its relevance to toxicity models in human [30,31]. Here, HEK 293T cells are seeded onto PPy substrates with prescribed unidirectional CNF at a dense 20-μm spacing, and cell cultivation for 1 and 3 days are shown in Figure 4b,c, respectively, similar to the culture period described before [32,33]. It is observed that cells on the aligned CNF show morphology characteristics of nanofiber-dependent orientation, i.e., a majority of the cells was dramatically influenced and elongated along the orientation of the CNF. When the CNFs were spaced more sparsely at 100 μm, cell shape and ordering were considerably less elongated, and a slight orientation is acquired as shown in Figure 4d,e. For the two different positioning densities with a controlled 20-μm and 100-μm spacing, respectively, cell spreading in preferential direction could be observed on parallel-aligned nanofibers, and the nanofiber alignment was capable of guiding cell extension, though cell orientation is noticeably less significant for the sparse 100-μm spacing. In contrast, HEK 293T cells seeded onto a nanofiber-free PPy substrate formed cells of isotropic, disordered orientation and polymorphic shapes, as shown in Figure 4f,g. Therefore, the enhancement of CNF alignment could have positive effects on cellular elongation behavior, possibly including cell spreading, as compared with nonuniformly distributed shapes of the nanofiber-free substrate [34,35].

**Figure 5:**
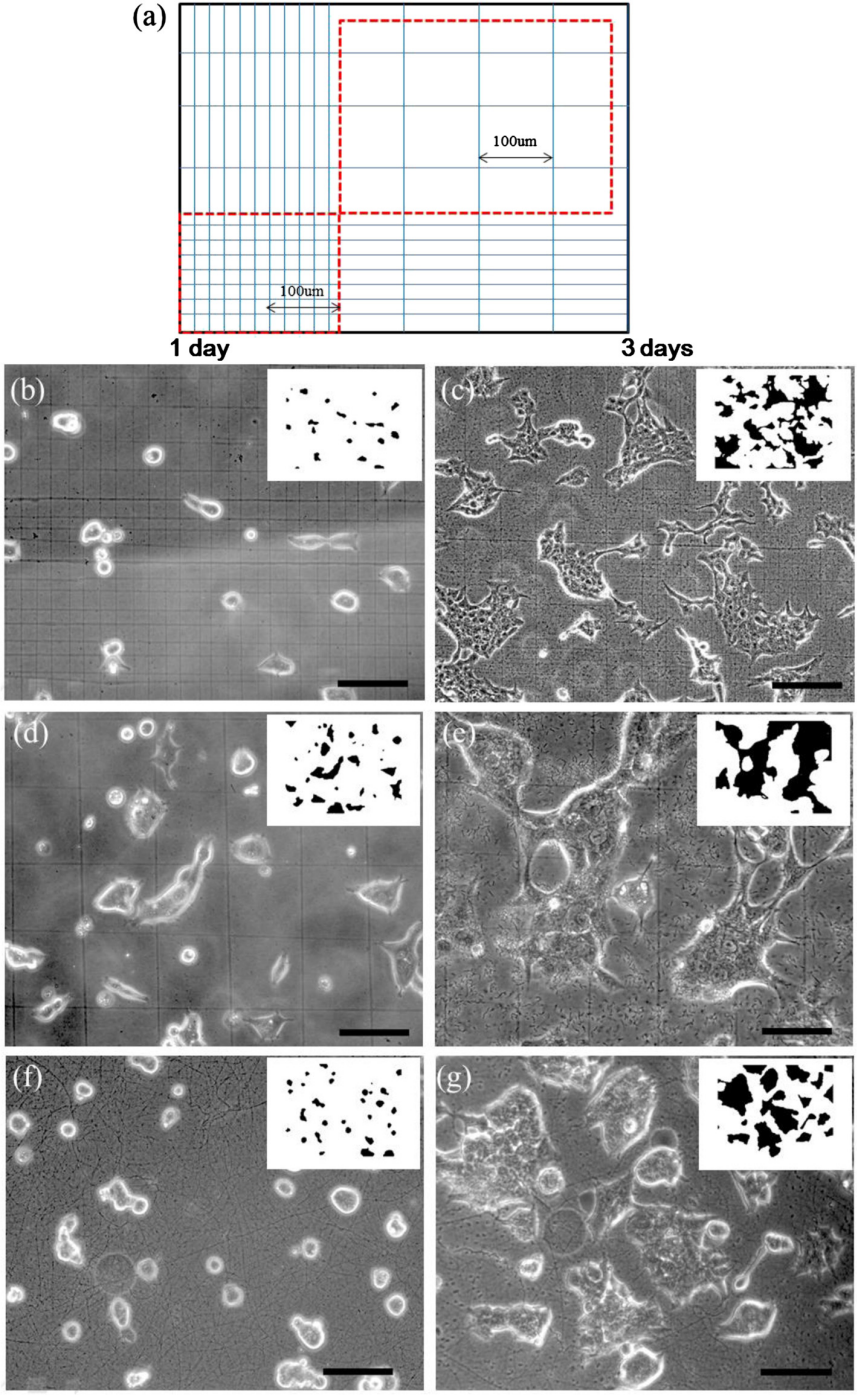
**OM images of HEK 293T cells seeded on the PPy substrate covered with aligned CNF**. (**a**) Schematic of the NFES grid-patterned CNF of different positioning densities. (**b**, **c**) Approximately 183 fibers/mm^2^ (20 μm), (**d**, **e**) approximately 37 fibers/mm^2^ (100 μm), and (**f**, **g**) cells seeded on randomly distributed CNF via conventional electrospinning. The smaller images at the right upper corner are shown to reveal the orientation of the cells (not on scale). The scale bars are 100 μm.

In Figures 4 and 5, the smaller images at the right upper corner are shown to reveal the orientation of the cells. Here the binary image analysis [36,37] of pixel counts for dark (D) and bright (B) regions are taken from the optical images of cells cultured for 1 and 3 days to account for cell spreading. In the binary processing, it should be noted that B region counts decrease and D region counts increase with the increase in cell spreading. A threshold value of 140 is used such that both B and D region counts have similar sensitivity over the positioning densities from parallel-aligned (10 to 50 fibers/mm^2^) and grid-patterned (37 to 183 fibers/mm^2^) CNF [38,39].

Figure 5a shows the schematic of the NFES CNF grid pattern at controlled 20- and 100-μm spacing, respectively. Qualitatively speaking, cell alignment revealed a relatively weak influence of the positioning density of the CNF grid patterns on cell shape and ordering. It is observed that no distinct elongated shape in cell morphology between the dense grid about 183 fibers/mm^2^ (Figure 5b,c), the sparse grid about 37 fibers/mm^2^ (Figure 55d,e), and randomly distributed mat (Figure 5f,g). However, the cells do exhibit confluence to some degree such that the dense CNF grid and randomly distributed mat seem to provide a specific contact guidance and oriented growth to the cells to result in spontaneously contracting cultures [39]. The confluence and contracting cultures are less significant in the sparse grid. We experimentally observed that CNF with distinct patterns, such as aligned or grid configurations, could have a significant impact and control the cell spreading in a different perspective.

### Relation between cell spreading and positioning density of CNF

**Figure 6:**
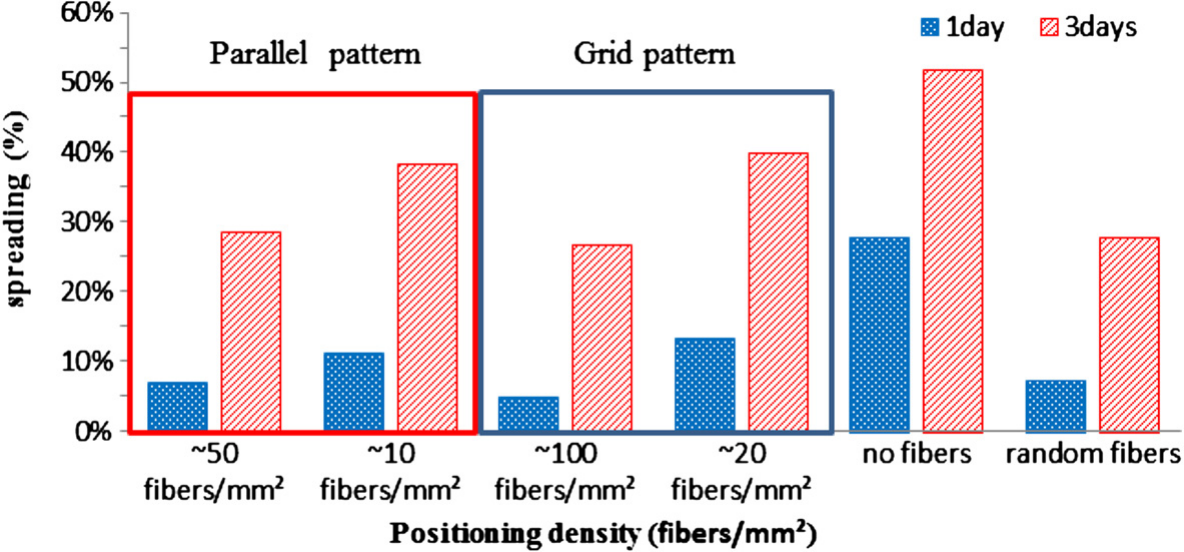
Quantification of cell spreading effect on different positioning densities of fibers for parallel and grid patterns.

Figure 6 shows the relation between cell spreading and different positioning densities using a binary image method as reported previously [36,37]. Cell viabilities and spreading after culture for 1 and 3 days with various positioning densities of CNF are illustrated. There were slightly more cells adhered to the sparse positioning density than the dense positioning density after cell seeding for 1 day, irrespective of parallel or grid pattern. The spreading of cells on the sparse positioning density dramatically increased compared to that on the dense positioning density after 3 days of culture. From the data obtained after 3 days of culture, cell spreading on sparse positioning density was faster than that on dense positioning density, which indicates that dense CNF could provide contact guidance and prevent cells from spreading. Similar trend of contact guidance can be observed for the case of randomly distributed CNF fabricated by conventional electrospinning method. Quantification results indicate cell spreading of 38.38% and 39.89% for the parallel pattern with approximately 10 fibers/mm^2^ and grid pattern with approximately 37 fibers/mm^2^, respectively, as compared with 27.71% for the randomly distributed CNF and approximately 51.73% for the nanofiber-free substrate. In the case of the dense grid pattern with positioning density of approximately 183 fibers/mm^2^, the smallest cell spreading is observed at 26.67%; comparable result is also found for the case of the parallel pattern with approximately 50 fibers/mm^2^ with 20-μm spacing wherein the cell spreading is 28.42%. It is conjectured that not only the density, but also spacing in CNF, is the main limiting factor to control cell spreading.

### Degree of HEK 293T alignment as judged by FFT

**Figure 7:**
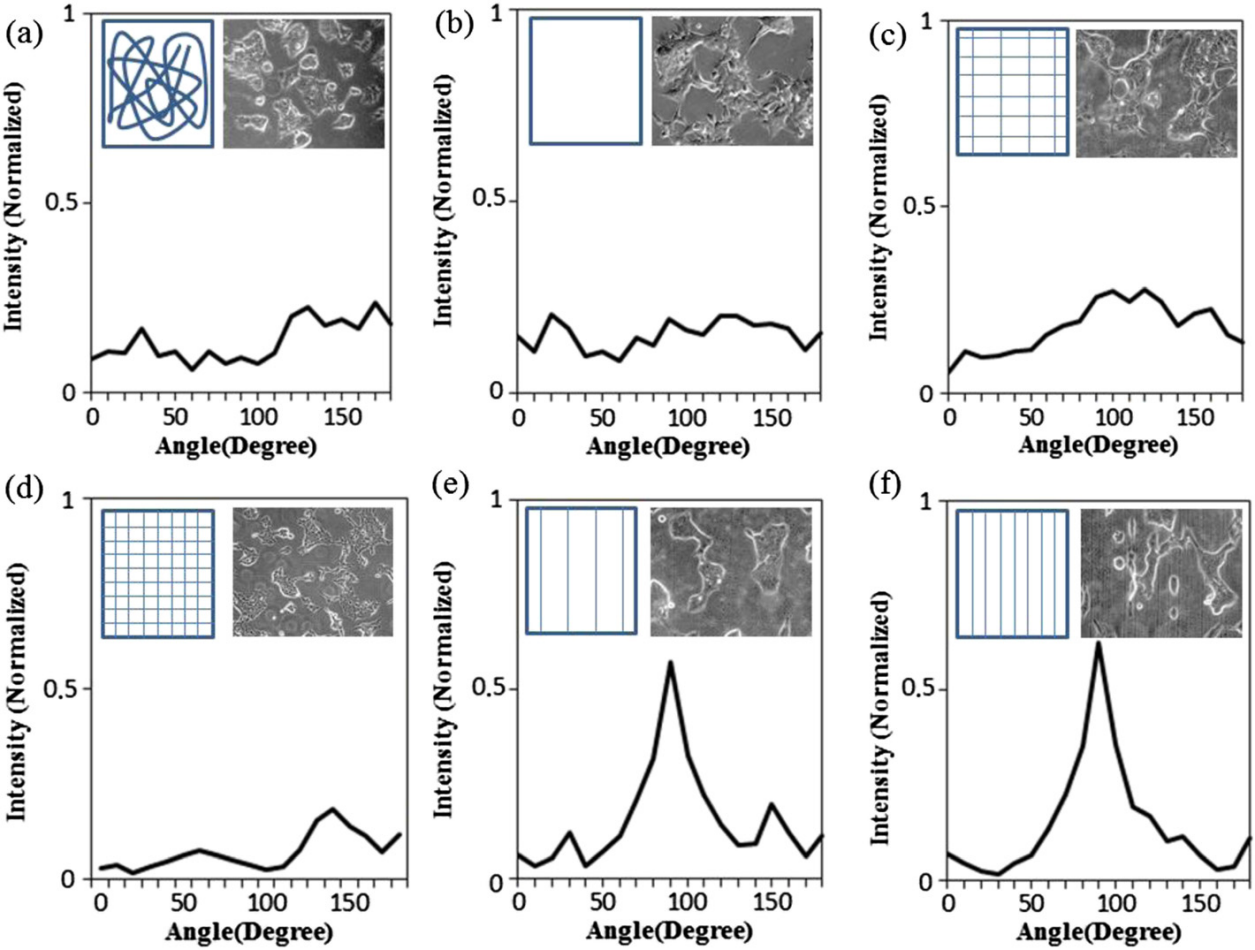
**FFT analysis of HEK 293T alignment as a function of CNF positioning density.** (**a**) On the substrate covered with randomly distributed nanofibers, (**b**) on the nanofiber-free solid substrate, (**c**, **d**) on PPy substrate covered with aligned grid patterns of CNF at different positioning densities, and (**e**, **f**) on PPy substrate covered with aligned CNF at different positioning densities for parallel patterns.

In order to quantify the effect of CNF on HEK 293T alignment and to characterize the degree of structural anisotropy, FFT analysis was applied and presented in Figure 7. It was previously demonstrated that aligned cell growth of cardiac tissue cardiomyocytes can be characterized by elongation of the cell shape supported by FFT data [20]. FFT analysis was carried out systematically in the following steps. First, an original data image containing cell shape is used to generate an output image of pixels distributed in a symmetrical, circular shape. Theoretically, this frequency distribution at specific pixel intensities in the data image should be identical in any direction. Therefore, the distribution of the angles at which cells were arranged in the analyzed images can be obtained by summation of Oval Profile similar to [20]. It is reported that the sharper and higher the peak, the more precisely the CNFs were aligned along a specific axis of orientation [40]. Experimentally, no overt peak can be observed for the cells on randomly oriented CNF, and the random distribution of cells is confirmed in Figure 7a. Similar observation can be found in Figure 7b, in which the cells were seeded on CNF-free PPy substrates, and no overt peak was produced in the FFT data, which was obviously related to the random distribution of cells. Figure 7c,d shows the grid patterns with 20- and 100-μm spacing, respectively. As anticipated, there was no overt peak produced in the FFT data, which was experimentally observed for the well-aligned grid patterns of cells. Presumably the grid patterns are thought to be able to limit the spreading of cells, which were not consistently obtained in our experiments, especially for the sparse grid with approximately 37 fibers/mm^2^. In contrast, parallel CNF indicates that the FFT alignment values sequentially increased as a function of positioning density (Figure 7e,f). Incrementally more aligned cells were closely related to the increasing of CNF positioning densities. Finally, Figure 7f indicates the highest degree of cell alignment and, most of the cells are nearly parallel.

## Conclusions

In this study, we utilized NFES to prepare CNF in a direct-write manner and deposit prescribed patterns of different positioning densities. The cell ordering and alignment of HEK 293T was grown on PPy substrate with CNF of different orientations and positioning densities. Our experiments showed that the presence of parallel-aligned CNF greatly influenced cell shape.

## Abbreviations

APS: Ammonium persulfate; CNF: Chitosan nanofiber; DMSO: Dimethylsulfoxide; FFT: Fast Fourier transform; NFES: Near-field electrospinning; PEO: Poly (ethylene oxide); PPy: Polypyrrole; PVA: Poly (vinyl alcohol)

## Competing interests

The authors declare that they have no competing interests.

## Authors' contributions

YKH designed the experiments, analyzed the data, and wrote the paper. SZC performed the experiments. ZYH helped in the revisions of the manuscript and preparation of response letters. All authors discussed the results, commented on, and approved the final manuscript.
